# Assessing the Toxicity of Metal- and Carbon-Based Nanomaterials In Vitro: Impact on Respiratory, Intestinal, Skin, and Immune Cell Lines

**DOI:** 10.3390/ijms252010910

**Published:** 2024-10-10

**Authors:** Juliana Carrillo-Romero, Gartze Mentxaka, Adrián García-Salvador, Alberto Katsumiti, Susana Carregal-Romero, Felipe Goñi-de-Cerio

**Affiliations:** 1 GAIKER Technology Centre, Basque Research and Technology Alliance (BRTA), 48170 Zamudio, Spain; carrillo@gaiker.es (J.C.-R.); mentxaka@gaiker.es (G.M.); garciaad@gaiker.es (A.G.-S.); katsumiti@gaiker.es (A.K.); 2 Center for Cooperative Research in Biomaterials (CIC biomaGUNE), Basque Research and Technology Alliance (BRTA), 20014 San Sebastián, Spain; scarregal@cicbiomagune.es; 3 CIBER de Enfermedades Respiratorias (CIBERES), 28029 Madrid, Spain; 4 Ikerbasque, Basque Foundation for Science, 48013 Bilbao, Spain

**Keywords:** nanotoxicology, industrial nanomaterials, cytotoxicity, oxidative stress, inflammatory response, biological barriers, in vitro assays, health effects

## Abstract

The field of nanotechnology has experienced exponential growth, with the unique properties of nanomaterials (NMs) being employed to enhance a wide range of products across diverse industrial sectors. This study examines the toxicity of metal- and carbon-based NMs, with a particular focus on titanium dioxide (TiO_2_), zinc oxide (ZnO), silica (SiO_2_), cerium oxide (CeO_2_), silver (Ag), and multi-walled carbon nanotubes (MWCNTs). The potential health risks associated with increased human exposure to these NMs and their effect on the respiratory, gastrointestinal, dermal, and immune systems were evaluated using in vitro assays. Physicochemical characterisation of the NMs was carried out, and in vitro assays were performed to assess the cytotoxicity, genotoxicity, reactive oxygen species (ROS) production, apoptosis/necrosis, and inflammation in cell lines representative of the systems evaluated (3T3, Caco-2, HepG2, A549, and THP-1 cell lines). The results obtained show that 3T3 and A549 cells exhibit high cytotoxicity and ROS production after exposure to ZnO NMs. Caco-2 and HepG2 cell lines show cytotoxicity when exposed to ZnO and Ag NMs and oxidative stress induced by SiO_2_ and MWCNTs. THP-1 cell line shows increased cytotoxicity and a pro-inflammatory response upon exposure to SiO_2_. This study emphasises the importance of conducting comprehensive toxicological assessments of NMs given their physicochemical interactions with biological systems. Therefore, it is of key importance to develop robust and specific methodologies for the assessment of their potential health risks.

## 1. Introduction

Nanotechnology has grown exponentially over the last few decades thanks to the unique properties of nanomaterials (NMs), which can enhance mechanical, optical, chemical, and electrical characteristics in diverse products [1]. Based on their structural composition, NMs are classified into organic, inorganic, and carbon-based NMs [2]. This work focuses on the latter two due to their wide application in different industrial sectors. Inorganic NMs, mainly based on metal and metal oxide, exhibit properties such as ultraviolet radiation absorption and catalytic, thermal, and antibacterial activity [3,4,5]. Among carbon-based NMs, carbon nanotubes are one of the most common, characterised by a high specific surface area, electrical conductivity, flexibility, and optical transparency [6]. These properties have enabled beneficial impacts across various industries, particularly in biomedicine, cosmetics, and food industries [7,8,9,10,11].

In biomedicine, NMs are distinguished by their applicability in areas such as diagnostics or drug delivery systems [10]. Among them, silica- and zinc-based NMs have been used as contrast agents in biomedical imaging systems [12,13]. Additionally, titanium oxide NMs and carbon nanotubes have been used in drug delivery and cancer therapy [14,15,16]. Cerium-based NMs are of particular interest because of their antioxidant and anti-inflammatory capacity, e.g., in the prevention of blindness or age-related macular degeneration [17,18]. In the cosmetics industry, NMs are mainly employed as active ingredients, carrier vehicles, and formulation aids [19]. Among them, titanium and zinc-based NMs are commonly found in sunscreens, acting as inorganic filters against ultraviolet radiation. Furthermore, silver NMs, due to their antibacterial and antifungal properties, are incorporated into soaps and anti-acne creams [20,21]. In the food industry, NMs improve food quality, safety, and functionality. For example, silver and carbon nanotubes NMs contribute to lighter, stronger, and less permeable packaging [22]. Additionally, zinc and titanium-based NMs extend the shelf life of food products through their antimicrobial properties.

However, the growing use of NMs has raised concerns about potential human health risks due to increased exposure [23,24,25,26]. While the larger surface area/volume ratio of NMs enhances catalytic activity, it may also lead to higher oxidation levels and cell signalling alterations [27,28,29]. A critical factor in assessing NM toxicity is understanding the route of exposure, which can occur through inhalation, ingestion, or skin contact [30]. Once internalised, NMs may be transported to different organs via the circulatory system. In the lungs, NMs can interact with the lung barrier and different cell types, leading to oxidative stress, inflammation, fibrotic processes, and genotoxicity, potentially leading to carcinogenicity [31,32,33,34]. Through ingestion, NMs may interact with the gastrointestinal barrier, leading to health disorders such as altered mucus secretion and intestinal permeability, as well as dysbiosis in gut microbiota [35,36]. When NMs penetrate the skin, smaller particles (<4 nm) can easily pass through healthy skin, while larger ones may only enter through damaged skin. Moreover, positively charged NMs are more likely to be internalised than negatively charged NMs, leading to inflammation, DNA damage, oxidative stress, and apoptosis [37,38,39].

To assess these effects in the respiratory, gastrointestinal, and dermal systems, various in vivo and in vitro methods have been used to understand the interaction between NMs and target systems. However, due to ethical and economic reasons, in vitro methods offer advantages, such as lower costs and faster toxicity screening [40]. Among them, cell viability assays are the most commonly employed for estimating cytotoxicity, using tetrazolium salts-based assays and dyes, such as MTS and Neutral Red and Alamar Blue assays [41,42,43,44].

Another key factor is the detection of reactive oxygen species (ROS) produced after NM exposure, which can disrupt various cellular processes [45,46,47,48]. ROS overgeneration can be quantified using ROS-sensitive dyes, such as DCFH [49,50].

Inflammatory processes, a typical NM toxicity mechanism, involve a signalling cascade leading to cytokine production, which is mainly quantified using ELISA kits [51].

It is also essential to evaluate DNA and chromosomal damage following interaction with genotoxic compounds. One of the most widely used methods for its evaluation is the micronucleus assay, characterised by its comparative ease of determining the genotoxic effect after NM interaction [52].

Apoptosis and necrosis assays are also key indicators of NM-induced cellular toxicity. The Annexin-V-propidium iodide assay is commonly used to detect these processes across various NMs [44].

To address the necessity for a comprehensive assessment of the toxicity induced by diverse types of NMs in the respiratory, gastrointestinal, and dermal systems, considering their interaction with the immune system, we developed a battery of in vitro assays. These assays aim to determine the sensitivity of specific systems to different types of NMs, focusing on key toxicity parameters such as cytotoxicity, genotoxicity, ROS generation, apoptosis/necrosis, and inflammation. Prior to assessing these parameters, we conducted a thorough physicochemical characterisation (TEM and DLS analysis) of different NMs with distinct properties. These include four different metal oxide NMs based on titanium.

(TiO_2_ NMs; NM 101), zinc (ZnO NMs; NM 110), silica (SiO_2_ NMs; NM 200), and cerium (CeO_2_ NMs; NM 212); a metal NM based on silver (Ag NMs; NM 300 K); and a carbon nanotube NM (MWCNT NMs; NM 400), all widely used in various industrial sectors.

For this purpose, five cell lines were selected to represent different parts of the human organism: the dermal system (3T3 fibroblasts), the gastric system (HepG2 and Caco-2 epithelial cell lines), the pulmonary system (A549 epithelial cell line), and the immune system (THP-1 monocyte cell line).

## 2. Results

### 2.1. Characterisation of Nanomaterials

To determine if the NMs exhibited different behaviours when they were suspended in milliQ water (batch) or three different culture media (Dulbecco′s Modified Eagle′s Medium (DMEM), Minimum Essential Medium (MEM) and RPMI-1640), the hydrodynamic diameter average (Z-Ave) distribution of the NMs dispersed in these media was measured at 100 µg/mL (the highest tested concentration) at two different time points (0 h and 24 h) using DLS analysis (Table 1).

Among the results, NM 101, NM 110, NM 300 K, and NM 400 showed a tendency to aggregate in the different media used at both 0 h and 24 h. This result indicated the rapid formation of aggregates independently of the application time/period. NM 200 and NM 212 were the most stable, with a similar average hydrodynamic size at 0 h and 24 h, as well as when compared to the batch.

**Figure 1:**
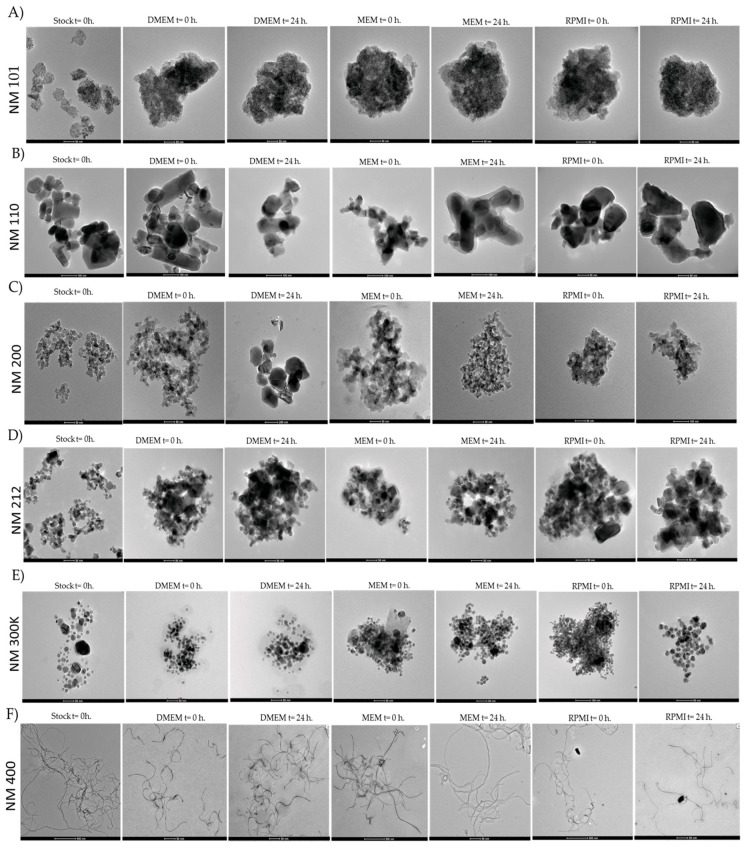
TEM images of (**A**) NM 101, (**B**) NM 110, (**C**) NM 200, (**D**) NM 212, (**E**) NM 300 K, and (**F**) NM 400 at 0 h in milliQ water (stock) and 0 h and 24 h in DMEM, MEM, and RPMI.

In addition, characterisation was carried out using Transmission Electron Microscopy (TEM) to evaluate the morphology of the NMs in stock suspensions at 0 h in milliQ water (batch measurement) and two different time points (0 h and 24 h) in three different cell culture media (DMEM, MEM, and RPMI). In the case of NM 101 and NM 200, a spherical or colloidal shape was observed. NM 110 showed a cubic, tetragonal, or orthorhombic morphology. For NM 212, an irregular shape was identified, whereas NM 300 showed round, triangular, or trapezium forms. Regarding NM 400, the shape was described as a straight-wall tube structure (Figure 1).

### 2.2. Cytotoxicity

Three different assays were employed to assess the cytotoxicity of the test samples: MTS assay, Alamar Blue (AB) assay, and Neutral Red (NR) assay (Table 2). Cell lines were exposed to five different concentrations of tested NMs for 24 h, with the lowest and highest concentrations being 1 µg/mL and 100 µg/mL, respectively.

Cytotoxicity results showed that in the case of NM 110, all the assays verified that the dermal 3T3 cell line was the most sensitive after exposure, showing an IC_50_ below 6 µg/mL (2.69 µg/mL in the MTS assay, 5.55 µg/mL in the AB assay, and 3.74 µg/mL in the NR assay) and an IC_80_ below 21 µg/mL (6.06 µg/mL in MTS, 20.24 in AB, and 17.30 in NR). In the case of NM 200, IC_50_ and IC_80_ values above 100 µg/mL were observed in all assays and all cell lines, except for the immune THP-1 cell line, whose IC_50_ was 35.91 µg/mL in the AB assay and 66.64 µg/mL in the NR assay. Based on NM 300 K results, all assays showed a higher affection in the gastrointestinal Caco-2 (IC_50_ below 20 µg/mL) and HepG2 (IC_50_ below 25 µg/mL) cell lines in MTS and AB assays. After exposure to NM 400, based on the IC_50_, there was a slight effect observed mainly after interaction with 3T3 and Caco-2 cell lines (IC_50_ of 69.38 µg/mL and 64.86 µg/mL, respectively). On the contrary, neither NM 101 nor NM 211 showed cytotoxicity at tested concentrations based on IC_50_ and IC_80_ results, both above 100 µg/mL in all cell lines.

### 2.3. Genotoxicity Assay

The genotoxicity of all NMs was determined using the comet assay. According to the results, none of the NMs caused genotoxicity in the Caco-2 cell line at the tested concentrations. The results are shown in the Supplementary Materials (Table S1).

### 2.4. ROS Production

**Figure 2:**
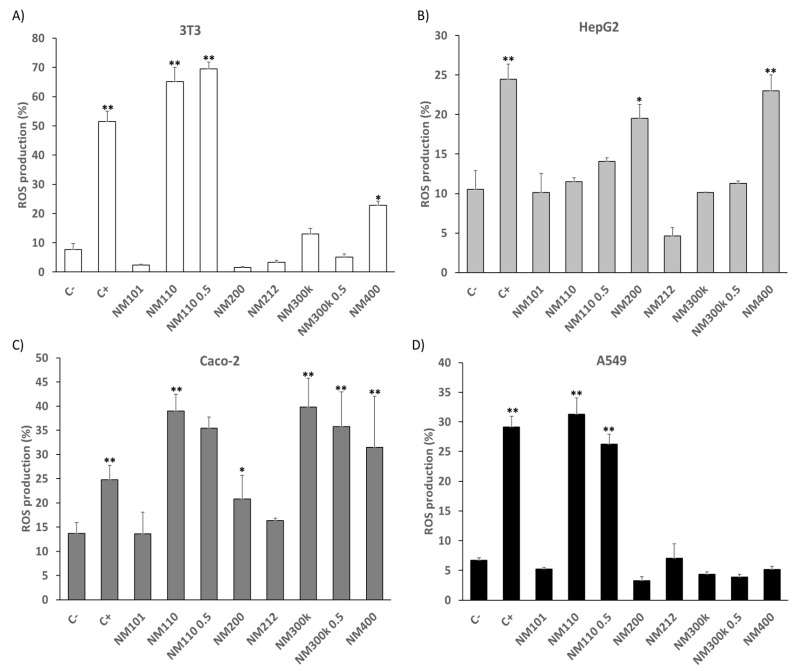
ROS production (%) of 3T3 (**A**), HepG2 (**B**), Caco-2 (**C**), and A549 (**D**) cell lines after exposure to all NMs (100 µg/mL and 50 µg/mL in NM 110 0.5 and NM 300 K 0.5) and to the negative (non-treated cells) and positive (H_2_O_2_) controls for 24 h. Results are expressed as means ± SD of six replicates per tested condition and three independent assays (*n* = 18). * Significantly different from negative control (C−) (*p* < 0.05). ** (*p* < 0.01).

Oxidative stress assessment based on DCFH assay with all NMs indicates variability with respect to the ROS generated in 3T3, HepG2, Caco-2, and A549 cell lines (Figure 2). In the case of the dermal cell line 3T3, a significant increase in ROS production was observed mainly after exposure to NM 110 and NM 110 0.5 (0.5 refers to exposure at 50 µM, rather than 100 µM, in contrast to the other particles) relative to the untreated control. With a lower significance, it can also be observed after NM 400 exposure (65.12 ± 4.92, 69.52 ± 2.32 and 22.8 ± 1.3, respectively) (Figure 2A). In the case of the gastrointestinal cell lines, HepG2 showed a significative increase in the case of the interaction with NM 200 (19.5 ± 1.76) and NM 400 (23.01 ± 2.03) (Figure 2B). With the same tendency, the Caco-2 cell line was also affected by NM 200 (20. 8 ± 4.86) and NM 400 (31.42 ± 10.58), as well as by NM 110 (39.01 ±3.45), NM 110 0.5 (69.52 ± 2.32), NM 300 K (39.81 ± 5.91), and NM 300 K 0.5 (35.74 ± 7.17) (Figure 2C). For the lung cell line A549, there was only a significant increase in ROS production by NM 110 (31.31 ± 2.71) and NM 110 0.5 (26.20 ± 1.68) (Figure 2D).

### 2.5. Necrosis/Apoptosis Assay

**Figure 3:**
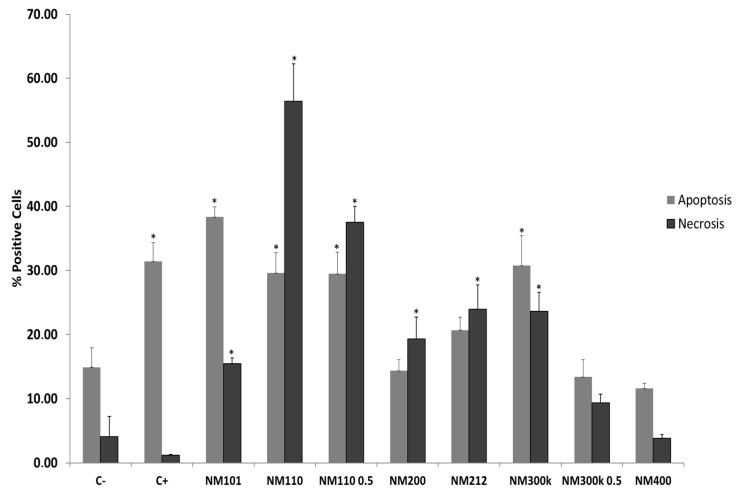
Necrosis/apoptosis (%) of THP-1 cell line after exposure to all NMs (100 µg/mL and 50 µg/mL in NM 110 0.5 and NM 300 K 0.5) and to the negative (non-treated cells) and positive (Camptothecin and SDS for apoptosis and necrosis, respectively) controls for 24 h. Results are expressed as means ± SD of six replicates per tested condition and three independent assays (*n* = 18). * Significantly different from negative control (C−) (*p* < 0.05).

Cell viability associated with NMs interaction was also assessed by investigating necrosis and apoptosis after 24 h of exposure to the THP-1 cell line. The percentage of positive cells for Annexin-V and propidium iodide (PI) in untreated and treated cells with all NMs, as well as the positive controls for apoptosis (Camptothecin) and necrosis (sodium dodecyl sulfate; SDS), are shown in Figure 3. Cells positive for Annexin V-Alexa Fluor (+) and negative for PI were considered apoptotic, while PI-positive cells were identified as necrotic.

After 24 h of incubation, treatment with NM 101, NM 110, NM 110 0.5, and NM 300 K showed a significant increase in both apoptotic and necrotic cells compared to the negative control. Specifically, the values for apoptotic cells with these NMs were between 29% and 39%, depending on the NM applied, and for apoptotic cells, they were between 15% and 56%. In the case of NM 200 and NM 212, the values for apoptotic cells after NM incubation did not show a significant increase relative to the negative control (14.35% in the case of NM200 and 20.68% for NM212), but for necrotic cells, there was a significative increase with respect to the untreated control (19.34% for NM 200 and 23.93% for NM212). In the case of NM 300 K 0.5 and NM 400, there was no significant difference between control samples and treated samples in either necrotic or apoptotic cells.

### 2.6. Inflammatory Response

**Figure 4:**
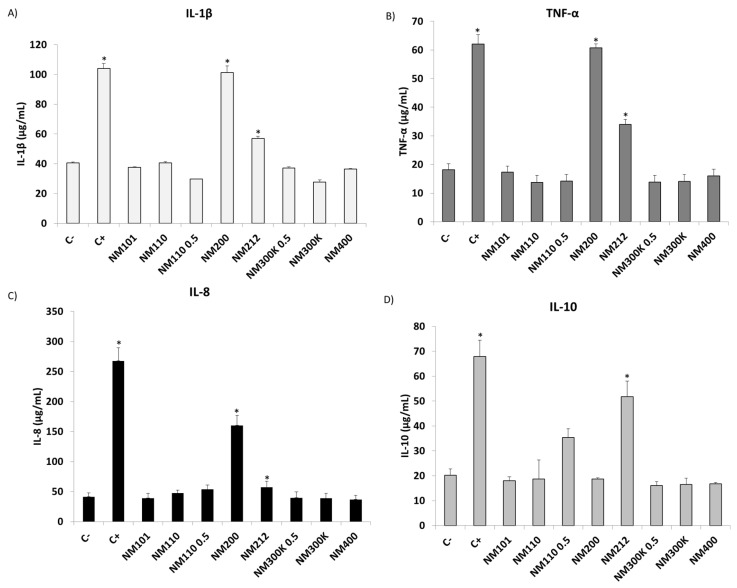
(**A**) IL-1β, (**B**) TNF-α, (**C**) IL-8, and (**D**) IL-10 release in THP-1 cell line after exposure to all NMs (100 µg/mL and 50 µg/mL in NM 110 0.5 and NM 300 K 0.5) and to the negative (non-treated cells) and positive (LPS) controls for 24 h. Results are expressed as means ± SD of six replicates per tested condition and three independent assays (*n* = 18). * Significantly different from negative control (C−) (*p* < 0.05).

Cytokine release (IL-1β, TNF-α, IL-8, and IL-10) triggered by the THP-1 cell line after incubation with all NMs was analysed using the ELISA assay (Figure 4). The results from the inflammation experiments showed that cytokine levels varied significantly with certain NMs after 24 h when compared to exposed and unexposed controls.

Following exposure to NM 200 and NM 212, there was a significant increase in the levels of the pro-inflammatory cytokines IL-1β (101.25 ± 4.51 μg/mL and 56.86 ± 1.66 μg/mL, respectively) (Figure 4A), TNF-α (60.77 ± 1.31 μg/mL and 33.98 ± 1.75 μg/mL, respectively) (Figure 4B), and IL-8 (160.31 ± 16.73 μg/mL and 57.02 ± 9.96 μg/mL, respectively) (Figure 4C) relating to the negative control. In contrast, there was no significant increase in these cytokines after interaction with the other NMs tested. In the case of the anti-inflammatory cytokine IL-10, a significant increase was observed only after interaction with NM 212 (51.70 ± 6.39 μg/mL) (Figure 4D).

## 3. Discussion

Assessing the toxicity of NMs to the human body is crucial, given their increasing use in various industries and their constant exposure in people’s daily lives. Despite the multiple benefits they offer, the relationship between their unique properties and potential adverse effects should not be underestimated. In this respect, NMs’ ability to penetrate biological barriers, such as the skin, respiratory tract, and gastrointestinal tract, and to interact with cells and tissues, possess health risks that need to be carefully assessed. These risks include a range of adverse health effects on various organs due to the generation of severe cellular injuries, such as cell membrane alterations, oxidative stress with the overproduction of ROS, inflammatory responses, and DNA damage [25,26]. This underlines the need for robust and specific assessment methods.

In this context, in vitro assays provide a valuable tool to examine these effects, allowing rapid and cost-effective screening. These methods are essential to understanding how the physicochemical characteristics of NMs, such as their size, shape, and surface composition, influence their biological behaviour and toxic potential. However, despite the advances made in understanding the toxicity-inducing potential of diverse NMs, a significant controversy persists regarding the extent of their toxicological impact. There is no consensus neither on the dose necessary to cause toxicity nor on the biological effects generated, and there is a lack of toxicological evaluation [53].

Therefore, it has been necessary to develop several studies to assess the potential toxicity of these materials, considering a thorough physicochemical characterisation of the NMs and an assessment of the impact on the organism. The behaviour of NMs in biological systems, e.g., through their interaction with proteins, enzymes, and DNA, varies with their composition and solubility. This variability is key in determining stability and toxicity, and it is necessary to consider that NMs can have different effects depending on the tissue in which they are found [8]. Therefore, our studies focus on the evaluation of the behaviour of several commonly used NMs and their toxicological impact in cellular models that represent different systems of the human body.

According to the data obtained after characterisation by DLS, alterations have been observed with respect to the stability of the suspended compounds in the different media. For TiO_2_, ZnO, Ag, and MWCNT NMs, an increase in the hydrodynamic diameter was observed independent of time (both at 0 h and 24 h), and there was an increase when comparing the batch in milliQ water with the different culture media (DMEM, MEM, and RPMI). This suggests that, depending on the biological environment and the physicochemical characteristics of NMs, they can easily interact with lipids, proteins, and peptides and may produce a protein corona (PC) on their surface. Proteins that associate with NMs can change their hydrodynamic size, shape, and charge, which can affect their distribution and uptake by cells, modifying their cytotoxicity and increasing oxidative stress production and inflammation [54]. Therefore, the fact that in vitro interaction with culture media containing different organic molecules (such as proteins) leads to an increase in particle size and, possibly, to the formation of a PC, allows us to foresee that this trend may also be followed in vivo. Hence, there will be an alteration in the interaction of the NM with the biological systems in vivo, which is an important factor to consider when assessing the possible toxicity generated by a given NM. In contrast, SiO_2_ and CeO_2_ NMs did not show variations when comparing the hydrodynamic diameter of the particles suspended in milliQ water with those in culture media, which is high (>250 nm) in both cases. For these NMs, agglomeration is generated regardless of the culture medium used since the size is larger than that reported for TEM dry samples, whose size ranges around 16 nm in the case of SiO_2_ NMs and 70 nm with CeO_2_ NMs, according to the literature [55]. Therefore, it is possible that with such NMs there is an absence of PC formation. Moreover, the absence of changes in size over time for such NMs indicates that once they are in contact with the biological fluids and a process of PC is generated, they will remain unchanged over time.

With respect to the zeta potentials for NM 101 (−46.54 mV), NM 110 (−23.86 mV), and NM 200 (−47.7 mV), which were obtained from the eNanoMapper database [56] and NM 212 (−33 mV) and NM 300 K (7.69 mV), they contrasted with literature ([57] and [58] respectively), which indicates the stability of the particles in the culture medium. NM 101, NM 200, and NM 212 demonstrated good stability, with values below −35 mV. However, there is a slight reduction in stability observed for NM 110, while NM 300 K displays a notable lack of stability. While the published data demonstrate overall stability, this is not reflected in the DLS results. It is therefore possible that the method of sample preparation differed slightly, resulting in a discrepancy between the two sets of data.

Moreover, it is important to note that DLS measurement is not recommended for carbon nanotubes, as it is widely accepted that DLS measures are based on the principle that the particles are spherical and well dispersed, factors that are not met in the case of this NMs [59]. We only employed these data to estimate the aggregate state over time in our research.

All these results indicate that when assessing the potential toxicity of an NM interacting with a specific organ, it is necessary to evaluate its capacity to generate a state of aggregation and PC. Therefore, in vitro assays are presented as very useful since the interaction between NMs and culture media containing proteins and other physiologic biological molecules is considered while assessing the target parameter.

Once the characterisation of the NMs in interaction with the culture media was carried out, different assays were performed with representative lines of the skin (3T3), the gastrointestinal system (Caco-2 and HepG2), the pulmonary system (A549), and the immune system (THP-1). Initially, different cytotoxicity assays based on colourimetry and fluorimetry were performed. Although the use of such assays to assess cytotoxicity is widely accepted, there is some concern that the intrinsic absorbance or fluorescence of NMs, as well as the interaction with dyes, may alter the results [60]. In this study, we were able to check that the cytotoxicity test performed (MTS, AB, and NR assays) did not generate any interaction between the compounds and the assay itself that could alter the results. It should be noted that an extra cytotoxicity assay, the lactate dehydrogenase (LDH)-based assay, was also carried out, but an interaction between the assay and the tested NMs was obtained, and therefore this assay was discarded for determining the potential cytotoxicity of the NMs. Subsequently, assays based on ROS detection (DCFH assay), apoptosis/necrosis (Annexin-V and PI), and inflammation (ELISA assays) were performed. These assays allow the analysis of such parameters with great sensitivity, indicating possible alterations or damage produced by NMs at the cellular and molecular level under subtoxic conditions.

Regarding the evaluation of the generation of toxicity by the NMs assessed on the skin, the cytotoxicity assays based on MTS, AB, and NR showed that the dermal line 3T3 has a high sensitivity to exposure to ZnO NMs, presenting the lowest IC_50_ values with respect to the rest of the cell lines (IC_50_ in all three assays below 6 µg/mL). Similarly, the ROS assay in 3T3 follows the same trend, with ZnO NMs generating the highest ROS production after exposure, both to the maximum concentration evaluated (100 µg/mL) and to half concentration (50 ug/mL), which was tested to check the effects generated at a sublethal concentration due to the high toxicity observed in the cytotoxicity assays. Several studies have evaluated the cytotoxic and ROS-producing capacity of ZnO NMs in skin cell lines. It has been shown in the literature that ZnO NMs present cytotoxic effects on different primary fibroblast cell lines (hPDLF and mDF) at concentrations ranging from 50 to 100 µg/mL, which agrees with the results obtained in this work [61]. Studies with HaCaT (immortalised human keratinocytes) and A375 (human skin melanoma cells) cells have similarly demonstrated a significant decrease in cell viability as well as an increase in ROS production following interaction with ZnO NMs [62,63].

Continuing in 3T3, both the AB and ROS assays show an increase in cytotoxicity (69.38 µg/mL) and in the percentage of ROS production (a significant increase with respect to C-; 22.8%) upon exposure to MWCNT NMs. These results agree with those obtained by Patlolla et al., who evaluated the cytotoxicity generated by MWCNTs in normal human dermal fibroblasts, concluding that cytotoxicity occurred after exposure to concentrations above 40 µg/mL [64]. Likewise, the absence of cytotoxicity and ROS production after exposure to TiO_2_, SiO_2_, CeO_2_, and Ag NMs is consistent with studies that have reported similar results [65,66,67,68].

Therefore, special attention needs to be paid to the effects of ZnO NMs on the dermal line since, as mentioned above, a wide variety of products used by people daily (such as sunscreens) contain such NMs. Consequently, the use of such products results in continuous and prolonged exposure over time in the human body by skin contact. Moreover, although the toxicity observed for MWCNTs is lower than for ZnO NMs, it is important to consider the sensitivity that they can generate when they meet the skin. It is crucial to remember that, in addition to their presence in products such as cosmetics or treatments for skin regeneration for the population, one of the main dangers of chronic exposure to these NMs is skin exposure in workers involved in their production.

The potential toxic effect of NMs in the gastrointestinal system has been investigated using the colorectal cell line Caco-2 and the liver line HepG2. This approach enables the characterisation of the potential alterations that may occur when a toxic NM reaches the epithelial barrier of the small intestine and subsequently, via the bloodstream, reaches the liver cells. Cytotoxicity assays have shown that Ag NMs display the highest cytotoxic effect in the gastrointestinal cell lines compared to the rest of the cell lines studied. In this case, the IC_50_ in the MTS and AB assays were between 4.45 and 18.32 µg/mL for Caco-2 and between 24.19 and 25.77 in the case of HepG2. Similar results have been described in the literature: in HepG2, a cytotoxic effect was observed after interaction with Ag NMs (IC_50_: 3.38 µg/mL obtained using the MTT assay) [69]. In the case of Caco-2, another study determined that a short-term toxic effect and ROS generation were generated by exposing them to Ag NMs with different surface coatings [70]. In both studies [60,61], the NR assay established an IC_50_ of 98.06 µg/mL for Caco-2 and 1.02 µg/mL for HepG2. It is possible that the difference in results using this type of assay when compared to the MTT and AB assay results may be because the inherent optical properties of Ag NMs may interfere with the fluorescence of the dye used to assess cytotoxicity in the NR assay [71].

For ZnO NMs, as in the case of the dermal line, high cytotoxicity is also generated in the liver and colon cell lines, although with IC_50_ values in all assays higher than those observed for 3T3 (between 15 and 22 µg/mL). For both cell lines, several studies have previously evaluated the cytotoxic effect of Zn NMs on Caco-2 and HepG2, concluding in both cases a size-dependent loss of cell viability (the smaller the size, the greater the cytotoxic effect) [72,73,74]. Cytotoxicity has also been reported in the Caco-2 cell line and other liver cell lines (HL-7702), comparing it with other NMs such as CeO_2_ [75]. Therefore, ZnO NMs are very likely to exhibit enhanced cytotoxicity due to the increased solubility of the compounds, causing the release of metal ions that are potentially more harmful when interacting with biological membranes.

In the case of NMs based on TiO_2_, SiO_2_, and MWCNT, an absence of cytotoxicity was observed with the evaluated assays (only an IC_50_ < 100 was obtained in the AB assay). Similar results have been previously shown for SiO_2_ NMs [76,77]. However, in the case of TiO_2_ and MWCNT NMs, there are contradictory studies in the literature about the toxicity they generate in gastrointestinal cell lines, which may be due to differences and changes in the physicochemical properties of the evaluated NMs when interacting with gastrointestinal cell lines. Furthermore, this effect could be increased in vivo after the digestion process, in which NMs are subjected to different alterations that could modify their intrinsic characteristics (agglomeration state, increased reactivity, or solubility) and, consequently, increase toxicity [78].

Regarding ROS production, Figure 2B,C show that Caco-2 and HepG2 display a significant increase in ROS production compared to the negative control after exposure to SiO_2_ and MWCNT NMs. Despite the cytotoxicity results not indicating a decrease in cell viability, the increase in ROS production shows that there is an increase in oxidative stress and thus sensitivity in the gastrointestinal cells. It is possible that the exposure time and concentration applied may not have been sufficient to overcome the threshold that triggers the cell death pathway [79].

Similarly, in Caco-2 cells, a significant increase is also observed after exposure to ZnO and Ag NMs, which agrees with the results obtained in the cytotoxicity assays. Because of the altered functionality of various cell organelles, an increase in ROS generation would consequently be accompanied by a decrease in cell viability. This phenomenon has already been described in the literature for other colon cell lines in interaction with these NMs [70,80].

Therefore, the results obtained indicate that it is necessary to pay special attention to the application of Ag and ZnO NMs in the development of products that involve the ingestion of these compounds. Furthermore, in addition to applying assays that consider the physicochemical characteristics of the particles and their possible interaction with the results obtained, it is essential to design in vitro tests that contemplate aspects such as digestion, since the modifications that may generate this process can lead to the development of an increase in toxicity that would not have been observed without the evaluation of these aspects.

With respect to the pulmonary track, cytotoxicity assays and ROS production assays in A549 cells did not show cell damage after TiO_2_, SiO_2_, CeO_2_, Ag, and MWCNT NM exposure, which is in line with previously published work on the sensitivity produced by these NMs in the A549 cell line [18,81,82,83,84]. However, our study has shown an increased sensitivity to interaction with ZnO NMs, similar to that observed in the rest of the cell lines studied. Several assays in other works were carried out after exposure of ZnO to lung cell lines, concluding that there is an increase in sensitivity, which leads to a decrease in cell viability and an increase in ROS production [85,86,87,88].

One of the main concerns about the toxicity exerted by ZnO NMs is that they are released in the form of aerosols after various manufacturing processes in the textile, rubber, or electronics industries, among others. Therefore, workers who are continuously exposed to these compounds can inhale and retain these NMs in the lungs [89]. Consequently, it is essential not to overlook how NMs affect respiratory systems. Despite the relatively low mass concentration of inhaled nanoparticles, their small diameter, enormous surface area, and potential for causing irreversible lung damage make them potentially harmful [90]. These findings suggest that prolonged exposure to certain NMs without the appropriate precautions could potentially result in lung diseases in humans.

Likewise, the process of interaction of NMs once they are introduced into the body and the immune response is crucial in assessing the toxicological capacity of these compounds. Initially, when NMs are presented as foreign bodies to immune cells in body fluids and tissues, the first cells to react are phagocytic cells, which are responsible for phagocytosing the particles. This process triggers the release of cytokines and chemokines to recruit neutrophils and monocytes and induce an inflammatory response. To evaluate this process, we selected the THP-1 cell line, a monocytic cell line differentiated into macrophages, which allowed us to assess the consequences of NM phagocytosis in vitro. For this purpose, in addition to the three types of cytotoxicity assays that allowed us to determine lethal and sublethal exposure concentrations, an apoptosis/necrosis assay and an inflammatory response assay were performed.

The cytotoxicity assays established that after exposure to ZnO NMs, unlike the results obtained for the rest of the cell lines, only a decrease in the IC_50_ was observed with the MTS assay (50.55 µg/mL). In contrast, THP-1 is the only cell line affected by SiO_2_ NMs (IC_50_ < 100 µg/mL in the AB and NR assays). In the case of exposure to Ag NMs, only a low IC_50_ is observed by the NR assay (5.15 µg/mL). The rest of the NMs did not exert a cytotoxic effect.

The apoptosis/necrosis assay (Figure 3) indicates a significant increase with respect to the negative control in both necrosis and apoptosis values after exposure to TiO_2_, ZnO, and Ag NMs and necrosis values with SiO_2_ and CeO_2_ NMs.

Therefore, although cytotoxicity assays only establish a decrease in cell viability after exposure to SiO_2_ and ZnO NMs, necrosis/apoptosis assays indicate that cell death pathways are also initiated and, therefore, in addition to colourimetric cytotoxicity assays, more sensitive complementary tests need to be carried out.

The inflammatory response (Figure 4) assessed through the secretion of the pro-inflammatory cytokines IL-1β, TNF-α, and IL-8 indicates that a response occurs with all three types of pro-inflammatory cytokines after exposure to SiO_2_ mainly, and CeO_2_ to a lesser extent. Moreover, the assessment of IL-10 indicates a significant response in the case of CeO_2_ NMs. Previous studies have shown that interaction with SiO_2_ NMs leads to an inflammatory response by stimulating and secreting pro-inflammatory cytokines, which can lead to chronic inflammation, leading to the development of diseases such as cancer [91]. Correlating with the data obtained in the cytotoxicity assays (Table 2), it can be observed that THP-1 is the only cell line affected by the interaction with SiO_2_ NMs, which is consistent with a triggering of the inflammatory response. Therefore, it is essential to understand the responses caused by the immune system following interaction with these NMs to see the effects of continued exposure to these compounds. Regarding the results obtained with IL-10, it has been widely described in the literature that CeO_2_ NMs are able to scavenge the most harmful free radicals, acting as a potent antioxidant that would allow them to act as anti-inflammatory agents [92].

The results obtained indicate the necessity for further investigation into the capacity of SiO_2_ NMs to generate toxicity. The tests carried out indicate a cytotoxic effect and cell death, which consequently activates a pro-inflammatory signalling cascade reflected in the secretion of pro-inflammatory cytokines. Therefore, if the inflammatory state persists over time, it can lead to chronic inflammation, which may result in the generation of diseases in different organs of the individual. A study utilising a mouse animal model demonstrated that acute administration of SiO_2_ NMs resulted in the overproduction of inflammatory cytokines and oxidative damage in various organs, including the lungs, heart, and liver [93]. In addition, a recent study of workers with long-term exposure to SiO_2_ NMs in an industrial environment concluded that those with skin lesions had significantly higher levels of cytokines such as IL-8 and TNF-α after decades of exposure to SiO_2_ NMs [94]. Therefore, in consideration of the acute exposure data and its potential prolongation in individuals unavoidably exposed due to occupational activities, it is imperative to identify protective measures to minimise such exposure and prevent the development of disease in the future.

## 4. Materials and Methods

### 4.1. Characterisation of Nanomaterials

Six different NMs were evaluated: TiO_2_ NMs (NM 101), ZnO NMs (NM 110), SiO_2_ NMs (NM 200), CeO_2_ NMs (NM 212), Ag NMs (NM 300 K), and MWCNT NMs (NM 400). All NMs were synthesised in the laboratory and supplied by the NaNoREG consortium. They were fully characterised by the European Commission Joint Research Centre (JRC) [95]. All NMs were characterised by Transmission Electron Microscopy (TEM–JEM–2100F UHR, JEOL Ltd., Akishima, Tokyo, Japan) to evaluate the morphology of NMs in the stock suspensions at 0 h in milliQ water (batch measurement) and at two different time points (0 h and 24 h) in three different cell culture mediums: DMEM (D0819), MEM (M4655), and RPMI-1640 (R8758), all of them obtained from Sigma-Aldrich (St. Louis, MO, USA). For TEM analysis, samples were deposited onto conducting carbon-coated copper grids by spraying 1 g/L suspensions of particles using a homemade spraying tool. After complete drying, samples were stained with a 1% *w*/*v* uranyl acetate (Agar Scientific, Rotherham, UK) solution in water. Moreover, hydrodynamic size distribution of all NMs was performed on a Zetasizer Nano ZS instrument (Malvern Panalytical, Malvern, UK) via dynamic light scattering (DLS) analysis at 25 °C. The data acquisition was carried out using the automatic adjustment of position, attenuation, and measurement time. Measurements of NMs samples were performed at 100 µg/mL on 1 mL sample volume suspended in milliQ water and MEM, DMEM, and RPMI-1640. Measures were carried out at 0 h and 24 h to evaluate the agglomeration state of the nanomaterials in the different media. Samples were previously vortexed and sonicated according to the supplier’s instructions. Three independent experiments and three replicates for each experimental point were carried out.

### 4.2. Cell Culture

Murine fibroblast 3T3 cell line (86110401) and human hepatocarcinoma HepG2 cell line (85011430) were obtained from the European Collection of Authenticated Cell Cultures (ECACC; Salisbury, UK). Human monocytes THP-1 cell line (TIB-202), colorectal adenocarcinoma Caco-2 cell line (HTB-37), and alveolar epithelial carcinoma A549 cell line (CCL-185) were obtained from the American Type Culture Collection (ATCC; Wesel, Germany).

3T3 cell line was cultured in DMEM + 10% fetal bovine serum (FBS; Gibco Thermo Fisher, Waltham, MA, USA). HepG2 and A549 cell lines were cultured in MEM + 10% FBS. THP-1 cell line was cultured in RPMI 1640 containing HEPES 25 mM + 10% inactivated FBS (FBSi) + β-mercaptoethanol 0.05 mM (Sigma-Aldrich, M6250, St. Louis, MO, USA). Caco-2 was cultured in MEM + 20% FBS + 1% non-essential amino acid (NEAA) (Sigma-Aldrich, M7145, St. Louis, MO, USA) + pyruvate 1 mM. All culture media were supplemented with 1% penicillin–streptomycin (P/S) solution (Life Technologies, Carlsbad, CA, USA). Cells were incubated at 37 °C and 5% CO_2_, and the culture medium was renewed every 2–3 days until confluency.

### 4.3. Cell Viability Assay

Cell viability was assessed in 3T3, Caco-2, A549, HepG2, and THP-1 cell lines through the MTS, Neutral Red, and Alamar Blue assays. Initially, for all assays, cells were seeded at 10^4^ cells/well in 96-well plates and incubated for 24 h at 37 °C and 5% CO_2_. The day after seeding, cells were exposed for 24 h to different concentrations of NMs (1, 10, 25, 50, and 100 µg/mL) before assessing the cytotoxic effects. CdSO_4_ (Sigma-Aldrich, 383082, St. Louis, MO, USA) was used as positive control and non-treated cells in culture medium as negative control. Cell viability was expressed by the half-maximal inhibitory concentration (IC_50_) and 80% inhibitory concentration (IC_80_), both determined by GraphPad Prism (version 10.0.1). Three independent experiments and six replicates for each experimental point were carried out.

#### 4.3.1. MTS Assay

The MTS assay is an improved version of the MTT assay, offering various benefits. Unlike the MTT assay, the MTS assay does not require a volatile organic solvent to dissolve the formazan product. Additionally, reactions can be measured and then placed back into the incubator for continued colour development. In this study, after treatment with NMs, cells were washed with PBS. Then, the MTS reagent (CellTiter 96^®^ AQueous One Solution Cell Proliferation Assay; Promega, G3581, Madison, WI, USA) was used according to the manufacturer’s instructions. Briefly, the MTS solution was added at a 1/5 dilution in a final volume of 100 µL in culture medium to each well, and cells were incubated at 37 °C and 5% CO_2_ for 1 h. After incubation, absorbance was measured at 490 nm wavelength using a spectrophotometer reader (Varioskan Lux, ThermoFisher Scientific, Waltham, MA, USA).

#### 4.3.2. Alamar Blue Assay

After exposure period, cells were washed twice with PBS. Then, 200 µL of medium with Alamar Blue dye (alamarBlue™ Cell Viability Reagent, Invitrogen™, DAL1025, Waltham, MA, USA) at 10% is added to each well. After that, cells were incubated for 4 h at 37 °C and 5% CO_2_. At the end of incubation, fluorescence was read at λex = 530 nm and λem = 590 nm in the spectrophotometer reader mentioned before.

#### 4.3.3. Neutral Red Assay

After 24 h of treatment, cells were washed with PBS, and NRS (Neutral Red Solution (0.33%), Sigma-Aldrich, N2889, St. Louis, MO, USA) (10 µL/100 µL culture medium) was added to each well. Well plates were incubated for 2 h at 37 °C and 5% CO_2_. Then, NRS was removed from all wells and washed once with Hanks’ Balanced Salt Solution (HBSS, Sigma-Aldrich). After that, the Neutral Red Assay Solubilization Solution (N4395) was added to the cells to dissolve the NR in the lysosomes, and the plate was shaken in a plate shaker for at least 10 min. Finally, the absorption of light by the NR molecules within the cells was measured in the spectrophotometer at 540 nm wavelength.

### 4.4. ROS Detection Assay

The potential for oxidative stress induction by all NMs was assessed using the DCFH-dye (Sigma-Aldrich, D6883, St. Louis, MO, USA) in four different cell lines: 3T3, Caco-2, A549, and HepG2. Initially, cells were seeded in 24-well plates at a density of 10^5^ cells/well for 24 h. Then, cells were treated with all NMs at 100 µg/mL (50 µg/mL in the case of NM 110 0.5 and NM 300 K 0.5 as well) for 24 h. After that, cells were washed with PBS and exposed to DCFH-DA (50 µM) by adding it to Hank’s Balanced Salt solution (Sigma-Aldrich, H8264, St. Louis, MO, USA) at 37 °C for 30 min in darkness. Subsequently, cells were washed again, resuspended in PBS, and analysed in the spectrophotometer at λex = 504 nm and λem = 529 nm. H_2_O_2_ (Sigma-Aldrich, H1009, St. Louis, MO, USA) at 0.5 mM was used as positive control and non-treated cells as negative control. Three independent experiments and six replicates for each experimental point were carried out.

### 4.5. Apoptosis/Necrosis Assay

Apoptosis/necrosis analysis was assessed by Annexin-V (Invitrogen™, A13201, Waltham, MA, USA) and Propidium Iodide (PI) (Sigma-Aldrich, P4170, St. Louis, MO, USA) fluorochromes in THP-1 cell line by flow cytometry to measure early apoptotic event and necrosis, respectively. After NM exposure to 100 µg/mL (50 µg/mL in the case of NM 110 0.5 and NM 300 K 0.5 as well) for 24 h, cells were collected and washed with cold phosphate-buffered saline (PBS). Then, cells were re-centrifugated and washed with Annexin-V Binding Buffer (10 mM of HEPES, 140 mM of NaCl, 2.5 mM of CaCl_2_, and pH of 7.4) (Sigma-Aldrich, St. Louis, MO, USA). After that, cells were incubated with Annexin-V (5 µL/100 µL of cell suspension) for 15 min at room temperature in darkness. Then, cells were washed and treated with PI (1 µL/100 µL of cell suspension) just prior to analysis via flow cytometry (Beckman Coulter, Brea, CA, USA). Cells treated with Camptothecin (apoptosis) (Sigma-Aldrich, PHL89593, St. Louis, MO, USA) and SDS (SDS; Sigma-Aldrich, L3771, St. Louis, MO, USA) (necrosis) were used as positive control and non-treated cells as negative control. Three independent experiments and six replicates for each experimental point were carried out.

### 4.6. Genotoxicity Assay

The genotoxicity assay was carried out based on micronucleus assay (In vitro MicroFlow^®^ Micronucleus Analysis kit, Litron Labs Ref: In vitro–250/50) according to the manufacturer’s instructions manual. Briefly, Caco-2 cells were seeded at 2 × 10^5^ cells/mL in 12-well plates. After that, cells were treated with the particles for 48 h at five different concentrations (1 × 10^−4^, 1 × 10^−3^,1 × 10^−2^, 1 × 10^−1^ µg/mL). Subsequently, the process of fluorescent dye labelling was executed following the protocols provided by the kit by flow cytometry. Three independent experiments and six replicates for each experimental point were carried out.

### 4.7. Cytokine Production Determination

The extracellular release of four cytokines, IL-1β, IL-10, TNF-α, and IL-8, induced by all NMs, was assessed on THP-1 cell line using commercially available ELISA kits (Invitrogen™ KAC1211, KHC0101, KHC3011, and KHC0084, respectively, Waltham, MA, USA), following the manufacturer’s protocols. The expression levels were examined in the conditioned medium after cell exposure to 100 µg/mL (50 µg/mL in the case of NM 110 0.5 and NM 300 0.5 as well) for 24 h. After incubation, cell culture media were diluted at a 1:3 ratio in the assay diluent and subsequently incubated with conjugated cytokine antibodies. After several plate washing steps, tetramethylbenzidine substrate was added, and results were quantified in pg/mL, with absorbance readings taken in the spectrophotometer at λ = 450 nm. Lipopolysaccharide (LPS) (Sigma-Aldrich, L2630, St. Louis, MO, USA) was used as positive control at a final concentration of 1 μg/mL, and PBS was used as negative control. Three independent experiments and six replicates for each experimental point were carried out.

### 4.8. Statistical Analysis

The data were presented as means ± standard deviation. The Kolmogorov–Smirnoff test verified the data’s normality, and Levene’s test verified the variances’ homogeneity. ANOVA was used to evaluate group differences, and then a Bonferroni–Dunn post hoc test was performed. *p*-values less than 0.05 were regarded as statistically significant. For all analyses, GraphPad Prism 10 was used.

## 5. Conclusions

While NMs offer numerous advantages, their distinctive characteristics may also present considerable health hazards that necessitate meticulous assessment due to their increasing use in a variety of industrial sectors and their constant exposure in daily life. The capacity of NMs to traverse biological barriers and interact with cells and tissues can result in severe cellular injury, including the disruption of cell membranes, oxidative stress, inflammatory response, and DNA damage. This highlights the necessity for robust assessment methods.

The results obtained with representative cell lines from the skin, gastrointestinal tract, lung, and immune system determine that sensitivity varies depending on the NMs with which they interact. ZnO NMs have been demonstrated to exhibit high levels of cytotoxicity and ROS production in the skin and lung cell lines. MWCNTs showed the same trend in the dermal cell line but with a reduced effect. Cytotoxic effects of Ag and ZnO NMs and ROS production upon exposure to SiO_2_ and MWCNTs were observed in both gastrointestinal cell lines. In addition, ROS production by exposure to ZnO and Ag NMs was also observed in Caco-2 cells. Moreover, immune cells responded to SiO_2_ and CeO_2_ NMs with pro-inflammatory and anti-inflammatory effects, respectively.

It is evident that in vitro assays offer a valuable method for assessing these effects, providing a rapid and cost-effective practice for toxicity screening. These approaches are essential for understanding how the physicochemical characteristics of NMs influence their behaviour upon contact with biological systems. However, one of the limitations of this study is inherent to the use of in vitro assays, which do not fully reflect the complexity of in vivo conditions. To address this, the ongoing advancements in the development of more sophisticated in vitro models, such as co-cultures and 3D organotypic systems, are essential to enhance the correlation between in vitro and in vivo.

It is crucial to consider all these factors to determine the potential toxicity of NMs and to establish appropriate protective measures to minimise exposure and prevent the development of disease.

## Figures and Tables

**Table 1:** Hydrodynamic diameter average (Z-Ave) distribution of all NMs suspended in milliQ water (batch) at 0 h and three different culture media (DMEM, MEM, and RPMI) at two different time points (0 h and 24 h). Results are expressed as means ± SD of three replicates per tested condition and three independent assays (*n* = 9). * Statistically significant differences with respect to the batch measure (in milliQ water) at 0 h (*p* < 0.05).

Time Point (Hours)	Medium	NM 101 (TiO_2_ NM)	NM 110 (ZnO NM)	NM 200 (SiO_2_ NM)	NM 212 (CeO_2_ NM)	NM 300 K (Ag NM)	NM 400 (MWCNT NM)
		Z-Ave (d. nm) ± SD	Z-Ave (d. nm) ± SD	Z-Ave (d. nm) ± SD	Z-Ave (d. nm) ± SD	Z-Ave (d. nm) ± SD	Z-Ave (d. nm) ± SD
0 h	Batch	15.49 ± 0.22	23.63 ± 0.23	337.23 ± 9.83	415.6 ± 134.32	3.38 ± 3.35	1.08 ± 0.94
	DMEM	490.49 ± 27.49 *	240.77 ± 4.08 *	276.05 ± 6.96	311.57 ± 5.05	67.95 ± 0.92 *	611.58 ± 18.09 *
24 h	DMEM	472.02 ± 15.90 *	293.94 ± 8.11 *	258.26 ± 14.86	247.13 ± 3.53	74.12 ± 0.91 *	428.16 ± 25.65 *
0 h	Batch	16.97 ± 0.15	26.27 ± 0.28	350.45 ± 15.23	438.66 ± 79.13	1.3 ± 1.95	1.86 ± 1.06
	MEM	452.92 ± 8.34 *	267 ± 7.78 *	288.69 ± 12.07	337.51 ± 8.6	63.63 ± 0.81 *	518.8 ± 23.51 *
24 h	MEM	279.71 ± 5.34 *	224.85 ± 5.45 *	259.74 ± 32.50	274.24 ± 6.06	66.83 ± 1.53 *	312.88 ± 10.15 *
0 h	Batch	11.68 ± 3.23	23.66 ± 1.12	344.83 ± 24.46	431.07 ± 59.83	14.75 ± 7.95	1.86 ± 1.06
	RPMI	587.11 ± 16.31 *	289.09 ± 6.49 *	282.40 ± 11.25	291.54 ± 5.86	64.94 ± 2.65 *	546.78 ± 17.51 *
24 h	RPMI	278.88 ± 5.92 *	213.90 ± 4.93 *	223.69 ± 9.87	241.95 ± 5.46	53.55 ± 1.01 *	406.54 ± 61.07 *

**Table 2:** Cell viability (MTS, Alamar Blue, and Neutral Red assays) of 3T3, Caco-2, A549, HepG2, and THP-1 cells exposed for 24 h to NM 101, NM 110, NM 200, NM 212, NM 300 K, and NM 400. Results are expressed as IC_50_ and IC_80_ values (µg/mL). Results are expressed as means ± SD of six replicates per tested condition and three independent assays (*n* = 18). * Significantly different from negative control (C−) (*p* < 0.05).

		MTS Assay		Alamar Blue Assay		Neutral Red Assay	
NM Code	Cell Type	IC₅₀ (µg/mL)	IC₈₀ (µg/mL)	IC₅₀ (µg/mL)	IC₈₀ (µg/mL)	IC₅₀ (µg/mL)	IC₈₀ (µg/mL)
NM 101 (TiO_2_ NM)	3T3	>100	>100	>100	>100	>100	>100
	Caco-2	>100	>100	>100	>100	>100	>100
	A549	>100	>100	>100	>100	>100	>100
	HepG2	>100	>100	>100	>100	>100	>100
	THP-1	>100	>100	>100	>100	>100	>100
NM 110 (ZnO NM)	3T3	2.69 ± 0.32 *	6.06 ± 0.93 *	5.55 ± 0.39 *	20.24 ± 1.58 *	3.74 ± 0.35 *	17.30 ± 1.92 *
	Caco-2	15.04 ± 2.05 *	20.78 ± 1.66 *	14.93 ± 1.95 *	21.73 ± 2.17 *	14.23 ± 1.84 *	20.09 ± 2.65 *
	A549	16.39 ± 1.53 *	25.87 ± 2.35 *	64.65 ± 3.96 *	>100	14.55 ± 1.23 *	28.16 ± 2.42 *
	HepG2	19.48 ± 2.42 *	25.26 ± 2.19 *	17.24 ± 1.05 *	32.66 ± 1.97 *	17.10 ± 1.52 *	21.97 ± 1.04 *
	THP-1	50.55 ± 4.02 *	>100	>100	>100	>100	>100
NM 200 (SiO_2_ NM)	3T3	>100	>100	>100	>100	>100	>100
	A549	>100	>100	>100	>100	>100	>100
	Caco-2	>100	>100	>100	>100	>100	>100
	HepG2	>100	>100	>100	>100	>100	>100
	THP-1	>100	>100	35.91 ± 2.83 *	>100	66.64 ± 5.02 *	>100
NM 212 (CeO_2_ NM)	3T3	>100	>100	>100	>100	>100	>100
	Caco-2	>100	>100	>100	>100	>100	>100
	THP-1	>100	>100	>100	>100	>100	>100
	A549	>100	>100	>100	>100	>100	>100
	HepG2	>100	>100	>100	>100	>100	>100
NM 300 K (Ag NM)	3T3	91.21 ± 5.67	>100	>100	>100	>100	>100
	Caco-2	4.45 ± 0.53 *	60.39 ± 4.95 *	18.32 ± 2.05 *	>100	98.06 ± 6.53	>100
	A549	>100	>100	>100	>100	>100	>100
	HepG2	25.77 ± 2.05 *	>100	24.19 ± 2.23 *	43.98 ± 4.07 *	1.02 ± 0.13 *	>100
	THP-1	95.73 ± 5.93	>100	>100	>100	5.15 ± 0.98 *	>100
NM 400 (MWCNT NM)	3T3	>100	>100	69.38 ± 3.87 *	>100	>100	>100
	Caco-2	>100	>100	64.86 ± 5.62 *	>100	>100	>100
	A549	>100	>100	>100	>100	>100	>100
	HepG2	>100	>100	>100	>100	>100	>100
	THP-1	>100	>100	93.40 ± 6.85	>100	>100	>100

## Data Availability

The source data underlying the figures are available from the authors upon request.

## Supplementary Materials

The following supporting information can be downloaded at: https://www.mdpi.com/article/10.3390/ijms252010910/s1.
