# Supplementary material for: Assessing the Toxicity of Metal- and Carbon-Based Nanomaterials In Vitro: Impact on Respiratory, Intestinal, Skin, and Immune Cell Lines
Source: Int J Mol Sci. 2024 Oct 10;25(20):10910. doi: 10.3390/ijms252010910 (PMC11507852; doi:10.3390/ijms252010910)
Supplement: Supplementary file 1 [file ijms-25-10910-s001.zip › ijms-3234448-supplementary.pdf]

International Journal of *Molecular Sciences*

MDPI

Assessing the Toxicity of Metal- and Carbon-Based Nanomaterials In Vitro: Impact on Respiratory, Intestinal, Skin, and Immune Cell Lines

Juliana Carrillo-Romero [1,2], Gartze Mentxaka [1], Adrián García-Salvador [1], Alberto Katsumiti [1], Susana Carregal-Romero [2,3,4] and Felipe Goñi-de-Cerio [1,*]

1 GAIKER Technology Centre, Basque Research and Technology Alliance (BRTA), 48170 Zamudio, Spain
2 Center for Cooperative Research in Biomaterials (CIC biomaGUNE), Basque Research and Technology Alliance (BRTA), San Sebastián, 20014, Spain
3 CIBER de Enfermedades Respiratorias (CIBERES), Madrid, 28029, Spain
4 Ikerbasque, Basque Foundation for Science, Bilbao, 48013, Spain
* Correspondence: to whom correspondence should be addressed

Genotoxicity assay results

Table 1 shows results obtained after micronucleus assay with Caco-2 cell line after interaction with NM 110, NM 200, NM 300K and NM 400.

Table S1. Genotoxicity assay in Caco-2 cell line after exposure to NM 110, NM 200, NM 300K, and NM 400.

	Concentration (ug/mL)	MNs (fold increase)
Control	0.0000	1.00
NM 110	0.0001	1.03
	0.0010	1.24
	0.0100	1.04
	0.1000	0.76
NM 200	0.0001	0.86
	0.0010	0.90
	0.0100	1.17
	0.1000	0.76
NM 300K	0.0001	0.97
	0.0010	0.99
	0.0100	0.80
	0.1000	0.70
NM400	0.0001	1.08
	0.0010	1.08
	0.0100	1.08
	0.1000	0.85
